# Limited Sensitivity of a Rapid SARS-CoV-2 Antigen Detection Assay for Surveillance of Asymptomatic Individuals in Thailand

**DOI:** 10.4269/ajtmh.21-0809

**Published:** 2021-10-11

**Authors:** Anek Mungomklang, Nichapa Trichaisri, Jittima Jirachewee, Jaravee Sukprasert, Warut Tulalamba, Vip Viprakasit

**Affiliations:** ^1^ The Office of Disease Prevention and Control-Region 4, Department of Disease Control, Ministry of Public Health, Thai Government, Saraburi, Thailand;; ^2^ Samut Sakhon Provincial Public Health Office, The Office of Secretary-General, Ministry of Public Health, Thai Government, Samut Sakhon, Thailand;; ^3^ Siriraj Center of Research Excellence in Advanced Gene and Cell Therapy (Si-CORE-AGCT) and Thalassemia Center, Faculty of Medicine Siriraj Hospital, Mahidol University, Bangkok, Thailand;; ^4^ Division of Hematology/Oncology, Department of Pediatrics, Faculty of Medicine Siriraj Hospital, Mahidol University, Bangkok, Thailand

## Abstract

COVID-19 is an infectious disease caused by severe acute respiratory syndrome coronavirus 2 (SARS-CoV-2) and is a global pandemic. Therefore, rapid and accurate tests for SARS-CoV-2 screening are urgently needed to expedite disease prevention and control especially in community transmission. Since late December 2020, Thailand has faced a new wave of COVID-19 outbreaks. The Thai National Disease Control program at the Ministry of Public Health has identified suitable measure for mass screening. A SARS-CoV-2 antigen-based assay is a surveillance option for active cases. Here, we evaluated the feasibility and test performance of a rapid SARS-CoV-2 antigen test during our field activities in 1,100 asymptomatic individuals in Samut Sakhon, Thailand, during the second wave COVID-19 outbreak (December 26–30, 2020). The results showed that the rapid antigen test had a sensitivity of 47.97% (95% CI: 36.10–59.96%) and a specificity of 99.71% (95% CI: 99.15–99.94%) versus standard reverse-transcriptase polymerase chain reaction. The rapid test performed better in cases with higher viral loads determined by the cycle threshold value. In real-world setting, the test performance can be compromised by several factors including viral loads, logistic chains, temperature, technical expertise of the operators, validity, and accuracy of the testing itself. Our study highlights a prerequisite for reevaluation of any given testing before implementing it at the national level.

## INTRODUCTION

COVID-19 is caused by severe acute respiratory syndrome coronavirus 2 (SARS-CoV-2) and was first recognized in Wuhan, China, in December 2019; it is now a global pandemic.^1^ The WHO reports > 181 million confirmed cases in more than 200 countries leading to almost 4 million deaths (June 26, 2021; https://covid19.who.int).

Thailand is the first country outside China to report COVID-19 cases. These resulted from a Chinese tourist’s infection that subsequently spread to Thai citizens. Thus, Thailand has screened for infected individuals since the beginning of 2020.^2^ The first wave of the COVID-19 outbreak in Thailand spanned March and April 2020 with 3,310 COVID-19 patients by July 2020.^2^ During the first wave, pandemic control was based on testing patients under investigation (PUI) and their close contacts using the real-time reverse-transcriptase polymerase chain reaction (RT-PCR) detecting specific gene sequence of the SARS-CoV-2 virus as the gold standard technique for COVID-19 diagnosis.^3^^–^^5^ The Royal Thai government was able to successfully control the first wave of the outbreak after 2 months, and there was no report of any local transmission for more than 200 days.^2^^,^^6^

However, the tide turned in the second and third Thai waves, which emerged in December 2020 and are ongoing. The first cohort was found in migrant workers in Samut Sakhon province. Many infected individuals were later found throughout the country after having traveled. As of June 27, 2021, Thailand has 244,447 confirmed COVID-19 cases with 1,912 deaths; there are more than 3,000 cases daily via local transmission.^7^ This alarmingly high number of new domestic transmissions in Thailand has raised considerable concern and inevitably drives policymakers to revisit our prevention and control strategy.

The Thai Ministry of Public Health has initiated a comprehensive active case finding program at Samut Sakhon province since the beginning of this current outbreak. They focused our screening in camps of migrant workers, wet markets, and factories because these are vulnerable and economically disadvantaged populations with limited access to healthcare. However, standard RT-PCR requires at least 4 hours of laboratory time and skilled staff.^8^ Therefore, rapid screening tests with acceptable accuracy for SARS-CoV-2 are urgently needed to expedite disease prevention and control management in widespread community transmission. One possibility is the use of lateral flow immunoassay based on chromatography and antigen–antibody interactions—these assays are rapid and do not require any instrumentation.^9^

Data on the accuracy and reliability of lateral flow immunoassays and other rapid antigen test appear to be vary.^10^^–^^13^ The test sensitivity versus standard RT-PCR was reported to be high: 93.9% (95% confidence interval [CI]: 86.5–97.4%) in an early study using fluorescence immunochromatography (Ag-RDT, Bioeasy Biotechnology Co., Shenzhen, China).^12^ However, the sensitivities can vary even within the same kit, for example, 30.2% (95% CI: 21.7–39.9%) versus 50% (95% CI: 39.5–60.5%) using the dipstick immunochromatographic assay COVID-19 Ag Respi-strip (Coris BioConcept, Gembloux, Belgium).^10^^,^^13^ Mak et al. (2020) evaluated the BIOCREDIT COVID-19 Ag (BioVendor Research and Diagnostic Products, Brno, Czech Republic) and showed that the sensitivity values also varied (11.1–45.7%) depending on sample types.^11^ In Thailand, Chaimayo et al. studied the sensitivity of the Standard Q of COVID-19 Ag test (SD Biosensor, Chuncheongbuk-do, Republic of Korea) versus RT-PCR assay.^8^ They showed that the sensitivity of the rapid test is 98.33% (95% CI: 91.06–99.96%). However, a limited number of studies implemented this rapid antigen testing in a real-world screening setting. Therefore, it is critically important to evaluate feasibility in a real community outbreak before nationwide implementation.

Here, we report the field implementation of a rapid antigen screening program in migrant workers in Samut Sakhon province. This study evaluated the sensitivity and accuracy of SARS-CoV-2 rapid antigen tests compared with the gold standard molecular assay. Our data could help our health policy makers select the best suitable model for the COVID-19 disease control.

## MATERIALS AND METHODS

### Study setting and population.

We conducted a cross-sectional study in 1,100 asymptomatic migrant workers from Samut Sakhon province through our active case-finding program during the SARS-CoV-2 outbreak in Thailand between December 26–30, 2020. The local ethical committee at the Office of Disease Prevention and Control-Region 4, Department of Disease Control, Ministry of Public Health, Thai Government, Saraburi, Thailand, approved our study in accordance with the Helsinki Declaration.

### Clinical specimens.

Nasopharyngeal swabs were collected and kept in 2 mL of viral transport media (VTM). This media contains Hanks’s balanced salt solution, 0.4% fetal bovine serum, HEPES, and antibiotic and antifungal agents. This was stored at 2–8°C until testing by real-time RT-PCR assay at the National Institutes of Health of Thailand, Department of Medical Science, Ministry of Public Health, Nonthaburi, Thailand.

### Viral RNA extraction.

Here, 200-µL nasopharyngeal swabs were used to extract SARS-CoV-2 RNAs using the GenTi 32 Ultimate Flexible Automatic Extraction System (GeneAll Biotechnology, Seoul, South Korea). Extraction was performed according to the manufacturer’s instructions. Viral RNA was eluted with 100 µL buffer and used for the RT-PCR assay.

### SARS-CoV-2 RNA detection using real-time RT-PCR assay.

The COVID-19 RT-PCR reagent kit was obtained from the Department of Medical Sciences and targets the envelope gene (*E gene*) of *Sarbecovirus* as well as the Open Reading Frame 1ab (*ORF1ab*)* gene* according to the manufacturer’s instruction. A final reaction volume of 20 µL containing 5 µL of the RNA template; 5 µL of 4X CAPITAL qPCR Probe Mix, primers, and probe; 6.3 µL of enhancer mix (30% Tween 20 + 50% glycerol); and 1 µL of 20X RTase with RNase inhibitor. The TaqMan probe was used in these reactions. All reactions were performed on a CFX96 Touch instrument with T1000 Thermocycler (Bio-Rad, Hercules, CA) to amplify. The following cycling conditions were used: a cDNA synthesis step (30 min at 50°C), a hold step (1 min at 95°C), and 45 subsequent cycles of denaturation (15 s at 95°C) and annealing/elongation (45 s at 55°C). The result was interpreted by using Seegene Viewer (Seegene, Seoul, Korea) in which a cycle threshold value (Ct value) < 40 for all target genes was defined as a positive result; a Ct value > 40 were defined as a negative result.

### Rapid SARS-CoV-2 antigen detection assay.

The Standard Q COVID-19 Ag test (SD Biosensor, Chuncheongbuk-do, Republic of Korea) was used to detect SAR-CoV-2 nucleocapsid (N) antigen and tested in this study. This screening test is a rapid chromatographic immunoassay with a control line (C) and a test line (T). The control line region is coated with mouse monoclonal anti-chicken IgY antibody, and the test line region is coated with mouse monoclonal anti-SARS-CoV-2 antibody against SARS-CoV-2 N antigen. SARS-CoV-2 N antigen detection used mouse monoclonal anti-SARS-CoV-2 antibodies conjugated with colored nanoparticles. The antigen-antibody color particle complex migrates based on capillary force and is captured in the T region by the mouse monoclonal anti-SARS-CoV-2 antibody. The intensity of the colored test T line depends on the SARS-CoV-2 N antigen present in the sample.

According to the manufacturer’s instructions, 200 µL of each nasopharyngeal swab specimen was added to the provided extraction buffer. The filter nozzle cap was pressed tightly onto the extraction tube. Three drops of the extracted sample were added to the test device, and the test result was read in 15 to 30 min. Samples positive for COVID-19 antigen showed two colored lines with control (C) and test (T) lines. This evaluation was performed within 4 hours after the specimen reached the laboratory.

### Statistical analysis.

Demographic information was described via descriptive statistics. Continuous data were shown in mean, standard deviation (SD), median, and range. Categorical data were illustrated with numbers, percentages, and 95% CI. The test sensitivity and specificity were performed according to standard methods.^14^

## RESULTS

### Characteristic of immigrant workers from Samut Sakhon province during the second outbreak.

We registered 1,100 migrant workers from Samut Sakhon province with the second COVID-19 outbreak in Thailand. Their characteristics are shown in Table 1. The median age of the population is 33.39 years (range 13–60). The gender was 56.18% male and 43.82% female. Most subjects came from Myanmar (97.91%).

**Table 1:** Characteristics of 1,100 individuals during an active case finding campaign for COVID-19 control from the Samut Sakhon, Thailand

Characteristics	Results
Age (years), median (range)	33.39 ± 8.53 (13–60)
Gender	
Male	618 (56.18%)
Female	482 (43.82%)
Nationality	
Myanmar	1077 (97.91%)
Cambodia	13 (1.18%)
Laos	10 (0.91%)
Results of RT-PCR assay	
Positive	73 (6.63%)
Negative	1,027 (93.37%)
Ct value of *E* genes by RT-PCR	
Mean ± SD (min, max)	25.83 ± 5.58 (min 16.34, max 35.97)
Ct value of *Orf1ab* genes by RT-PCR	
Mean ± SD (min, max)	26.11 ± 5.57 (min 16.30, max 37.12)
Results of rapid antigen detection assay	
Positive	38 (3.45%)
Negative	1,062 (96.55%)

Ct = cycle threshold; RT-PCR = reverse-transcriptase polymerase chain reaction.

### Comparative study of real-time RT-PCR versus SARS-CoV-2 antigen assay

RT-PCR targeting the *E gene* and the *ORF1ab gene* of SARS-CoV-2 was implemented for SARS-CoV-2 RNA diagnosis. We found 73 positive cases (6.63%) from the 1,100 tested subjects. The average cycle threshold (Ct) values in COVID-19 positive cases of each gene are shown in Table 1.

We evaluated the performance of SARS-CoV-2 antigen detection, and the results were interpreted as positive when both the control (C) line and the SARS-CoV-2 antigen (T) line appeared within 30 minutes. The antigen results are shown in Table 2 compared with RT-PCR. This antigen detection kit’s overall sensitivity and specificity were 47.97% (95% CI: 36.10–59.96%) and 99.71 (95% CI: 99.15–99.94%), respectively. However, the sensitivity of the rapid test improved when we stratified the data based on the Ct: Ct < 20, Ct 20–30, and Ct > 30 cycles representing high, medium, and to low viral loading, respectively (Table 3). Nevertheless, the specificity remained as high at 99.71% across all Ct subgroups.

**Table 2:** Results of the Rapid SARS-CoV-2 antigen assay compared with RT-PCR analysis

Rapid SARS-CoV-2 antigen assay	Real-Time RT-PCR assay		
	Positive	Negative	Total
Positive	35	3	38
Negative	38	1,024	1,062
Total	73	1,027	1,100
Sensitivity (95% CI)	47.97% (36.10–59.96%)		
Specificity (95% CI)	99.71% (99.15–99.94%)		

CI = confidence interval; RT-PCR = reverse-transcriptase polymerase chain reaction; SARS-CoV-2 = severe acute respiratory syndrome coronavirus 2.

**Table 3:** The sensitivity and specificity of the Rapid SARS-CoV-2 antigen assay according to different viral loads based on Ct (cycle threshold) values

Rapid SARS-CoV-2 antigen assay	Real-time RT-PCR								
	(Ct < 20)			(Ct 21–30)			(Ct > 30)		
	Positive	Negative	Total	Positive	Negative	Total	Positive	Negative	Total
Positive	8	3	11	21	3	24	6	3	9
Negative	0	1,024	1,024	21	1,024	1,045	13	1,024	1,037
Total	8	1,027	1,035	42	1,027	1,069	19	1,027	1,046
Sensitivity (95% CI)	100% (63.06–100.00%)			50.00% (34.19–65.81%)			31.58% (12.58–56.55%)		
Specificity (95% CI)	99.71% (99.15–99.94%)			99.71% (99.15–99.94)			99.71% (99.15–99.94)		

CI = confidence interval; Ct = cycle threshold; RT-PCR = reverse-transcriptase polymerase chain reaction; SARS-CoV-2 = severe acute respiratory syndrome coronavirus 2.

## DISCUSSION

Thailand successfully controlled the outbreak during the first wave using the approach to prevent new cases by focusing on PUI and contact case tracing by RT-PCR to mitigate SARS-CoV-2 infection in the early stages of the pandemic.^2^ However, the second and third waves have presented increasing challenges, especially in congregate settings such as among migrant workers. Thus, rapid and deployable testing to screen thousands or hundreds of thousands of individuals is a key tool to control the pandemic.

Rapid detection of SARS-CoV-2 antigen is an attractive strategy because it can be performed at the point of care with results within 30 minutes.^8^^,^^11^^,^^12^ Like other commercially available rapid tests, these rapid assays can be manufactured at scale with low prices. They can be administered without any specific laboratory or instrumentation installed. Fast antigen testing has been accepted by the International Air Transport Association for a safe and efficient air travel.^15^^–^^17^ Passengers can use the negative result from recommended COVID-19 rapid antigen testing services as supporting evidence to onboard on many airlines and countries. For example, all air passengers arriving in the United States must show a negative COVID-19 result based on NAAT or rapid antigen test to prove recovery from COVID-19.^18^ Similar measures have also been used in Germany, China, New Zealand, among other nations. Moreover, rapid SARS-CoV-2 antigen test has been proposed for screening in asymptomatic people or high-risk congregate housing (such as nursing homes) as well as community settings such as prevention and control monitoring.^19^^,^^20^

Unfortunately, the use of rapid SARS-CoV-2 antigen testing in public health settings for surveillance in endemic locations remains rare. Rapid antigen testing is less sensitive than RT-qPCR and requires a higher viral load for SARS-CoV-2 detection.^21^^,^^22^ Bruzzone et al. recently showed rapid antigen tests for SARS-CoV-2 detection in a hospital setting: they had 78.7% and 100% sensitivity and specificity, respectively. These results were similar to those detected by RT-PCR. A Ct of 29 was defined as a cutoff value to maximize the detection in rapid antigen tests.^21^ The sensitivity of the rapid test was only 41.2%, and specificity was 98.4% versus RT-PCR results, whereas sensitivity was 100% compared with viral culture positivity.^23^

A recent review also highlighted considerable between-study variability in sensitivity and specificity estimates. These also varied by antigen testing kits and viral load of the sample.^24^ For instance, a subgroup analysis by viral load as defined by the Ct quantified a 53.8% absolute difference in sensitivity between samples with Ct 25 and > 25.^24^ The diagnostic performance of commercially available tests may differ substantially and primarily affect decision-making during test selection. The WHO recommends that SARS-CoV-2 antigen testing have a minimum of 80% sensitivity and 97% specificity.^9^ The European Center for Disease Control and Prevention (ECDC) has recently proposed a more conservative threshold of 90% for the sensitivity parameter especially in low-incidence settings.^22^ Therefore, the routine use of rapid antigen tests may be suitable in moderate-to-high disease intensity settings when high volumes of specimens are tested every day.

We evaluated the feasibility of using a SARS-CoV-2 rapid antigen test in a real-world setting from our active case finding scenario compared with a gold standard molecular assay via real-time RT-PCR method.^25^^,^^26^ Implementation of rapid antigen immunoassays can help accelerate screening at the population scale. Previously, the manufacturer of this antigen screening reported sensitivity and specificity of the Standard Q COVID-19 Ag test of SARS-CoV-2 were 96.52% (111/115, 95% CI: 91.33–99.04%) and 99.68% (310/311, 95% CI: 98.22–99.99%), respectively, using symptomatic cases from two trial sites in Brazil and India in 2020 (data available on the manufacturer instructions). The specificity of the SARS-CoV-2 N antigen monoclonal antibody was coated on a Standard Q COVID-19 Ag test developed from the WUHAN-01 strain; this strain is closely related to the SARS-CoV-2 strains detected previously in Thailand and relevant to our second outbreak individuals used in this study.^27^^,^^28^ Therefore, it is unexpected and disappointing to find a relatively low sensitivity of this screening test (47.97%) when applied in a real-world setting versus standard RT-PCR.

The differences in the test performance between laboratory/hospital-based and fieldwork settings could be due to several factors. One apparent reason is that in this active case finding, we performed the screening in asymptomatic individuals who might have low viral load representing various stages of SARS-CoV-2 infection.^29^ Nevertheless, some individuals with low Ct values (Ct < 20) can be detected with 100% sensitivity, which is closer to the validation study of the manufacturer (96.52%) in symptomatic patients.^30^ Moreover, several factors, such as variable clinical onset and immune status of the subjects, could play a role.^9^ In addition, the use of such a screening kit requires an appropriate logistic chain including temperature control for kit transportation, technical expertise of operators who must interpret the results on site, and the safety of handling contagious nasopharyngeal specimens.^19^^,^^31^ Recently, a publication demonstrated transportation and storage temperature also impaired the performance of the SARS-CoV-2 antigen test. Up to 11 commercially available SARS-CoV-2 antigen rapid tests were evaluated using different storage and operating temperatures as well as short- and long-term storage times. Their results indicated that higher temperatures (37°C) resulted in about 10-fold lower sensitivity of SARS-CoV-2 detection (eight of 11 kits). On the other hand, lower temperature (2–4°C) during storage would lower the specificity with a higher false-positive rate.^32^

Moreover, the validity and accuracy of any given screening test should be performed in various settings to recapitulate the heterogeneity of clinical settings from hospital-based work to fieldwork. There is clearly a vast difference in terms of viral and procedural factors. One cannot rely solely on a single study before it is adopted widely. Policymakers must use any new approach to slow the pandemic. Our evaluation is critical and complements the manufacturer’s data; both should be considered before implementing new technology in a real-world setting.

### Limitations of the study.

Due to logistical challenges, we could not perform direct preparation of the samples and testing for antigen detection immediately after sample collection. Therefore, we could not exclude the possibility that detection via this rapid test might lower leading to a lower sensitivity. We performed a rapid detection process using VTM collected in the laboratory within the four recommended hours—this time-lag was allowed based on the manufacturer’s guidelines. We have carefully controlled the VTM’s temperature during transport to preserve the viral integrity. Nevertheless, our study design could have minimized the effects of double samplings: one for VTM and one for rapid antigen testing. These could lead to differences in terms of sample quality and the starting amount of the viruses.

## CONCLUSIONS

The rapid assay for detection of SARS-CoV-2 antigen (Standard Q COVID-19 Ag test) was tested in an active case finding in Samut Sakhon province with 47.97% sensitivity (35/73; 95% CI: 36.10–59.96%) and 99.71% specificity (1,024/1,027; 95% CI: 99.15–99.94%) versus a standard RT-PCR. The sensitivity depends on the viral load as indicated by higher sensitivity in samples with low Ct values. Our results highlight challenges with antigen screening for SARS-CoV-2 infection between hospital-based and a real-world active case setting. New technology should be evaluated before nationwide implementation.
